# Antioxidant Activity of Aqueous Extract of Leaves and Seeds of *Datura metel* (Solanaceae) in Frog's Heart Failure Model

**DOI:** 10.1155/2022/5318117

**Published:** 2022-05-12

**Authors:** H. Mbida, D. E. Tsala, S. Aboubakar, S. Habtemariam, J. J. Edmond, E. F. Bakwo, J. Z. Minkande

**Affiliations:** ^1^Department of Biological Sciences, Faculty of Science, University of Maroua, P.O. Box 814, Maroua, Cameroon; ^2^Institute of Agricultural Research for Development, P.O. Box 2067, Yaoundé, Cameroon; ^3^Pharmacognosy Research Laboratories and Herbal Analysis Services, University of Greenwich, London, UK; ^4^Faculty of Medicine and Biomedical Sciences, University of Yaoundé I, P.O. Box 1364, Yaoundé, Cameroon

## Abstract

**Objective:**

The aim of this work was to evaluate the antioxidant potential of *Datura metel*.

**Materials and Methods:**

Heart failure was induced in the frog's heart by continuous perfusion of hydrogen peroxide. Survival time and some heart tissue parameters of oxidative stress were recorded in the presence of aqueous extracts of the leaves and seeds of *Datura metel*. Ascorbic acid was used as a reference drug.

**Results:**

H_2_O_2_-enriched Ringer's solution inhibited the negative inotropic and chronotropic effects of acetylcholine, indicating the desensibilization of muscarinic receptors due to H_2_O_2_-induced oxidative stress. These hearts had a relatively short survival time (14 minutes). In the presence of the aqueous extract of the leaves and seeds of *Datura metel* (1.5 and 2.5 mg/mL), the time necessary to cause the cardiac arrest was extended to 35 and 37 minutes, respectively, versus 29 minutes for ascorbic acid and 14 minutes for H_2_O_2_. Furthermore, antioxidant parameters (MDA, SOD, and CAT) were significantly improved in plant extract-treated hearts, compared to peroxidized hearts.

**Conclusion:**

Aqueous extract of the leaves and seeds of *D*. *metel* can extend heart survival time through antioxidant mechanisms.

## 1. Introduction

Oxidative stress is defined as an imbalance between prooxidants and antioxidants in favor of the former and is involving the production of reactive oxygen species (ROS) [1]. Under normal conditions, aerobic metabolism in mammals generates substances called ROS that are acting in small amounts in physiological processes [2]. However, excess production of ROS can become toxic to major components of the cell, including lipids, proteins, and nucleic acids and therefore leads to oxidative stress [3]. Oxidative stress is involved in various diseases such as cardiovascular diseases, cancer, diabetes, neurodegenerative diseases, and in the aging process [4]. The fight against ROS by organisms is usually provided by antioxidant systems that are synthesized by the body or provided by the diet. It has been shown that oxidative stress can be successfully induced by using hydrogen peroxide on cultured cells and in several animal models, particularly on the heart of amphibians [5, 6]. An antioxidant can be defined as any substance capable, at a relatively low concentration, of competing with other oxidizable substrates and thus lowering or preventing the oxidation of these substrates [7]. Many studies have shown that plants have antioxidant properties largely due to their phenolic content [2, 8]. Phenolic compounds, therefore, play an important role in human health because of their various pharmacological activities such as anti-inflammatory, antiallergic, antimicrobial, antiviral, anticancer, cardioprotective, and vasodilatory [9–11]. In addition, they can prevent oxidative modification by neutralization of free radicals, oxygen scavenging, or peroxide decomposition via their antioxidant activities [12–14].

*Datura metel* is a plant traditionally used in the treatment of asthma, convulsions, pain, and rheumatism and has hypolipidemic properties [15, 16]. Phytochemically, this plant is rich in phenolic compounds, alkaloids, glycosides, triterpenes, and flavonoids [17]. In a recent study, aqueous extracts of the leaves and seeds of a plant have shown cardiotonic activity, probably through cholinergic pathways [18]. The present research work was designed to assess a possible antioxidant activity of the same material in a heart failure model.

## 2. Materials and Methods

### 2.1. Reagent and Equipment

#### 2.1.1. Ringer's Solution for Amphibians

NaCl (9 g/L), KCl (0.42 g/L), CaCl_2_ (0.24 g/L), dextrose (1.0 g/L), and NaHCO_3_ (0.5 g/L), in 1 L of distilled water was prepared.

#### 2.1.2. Reagents

Acetylcholine, ascorbic acid, and hydrogen peroxide were used. Ascorbic acid and hydrogen peroxide were obtained in pharmacy and acetylcholine came from Sigma.

#### 2.1.3. Equipment Used

The Orchid Scientific brand Kymograph, model SRD-01, series SRD-01/17-18/39 made in India was used to record cardiac contractility parameters.

The centrifuge brand (UNIVERSAL 320R Hettich) was used for the centrifugation of homogenates and some other reaction media before reading the absorbance.

The spectrophotometer brand (Secomam, Prim Light, Prim Advanced) was used to read absorbance.

### 2.2. Plant Material and Extract Preparation

The leaves and seeds of *Datura metel* were harvested early in the morning from a flowery plant in the locality of Koza (Koza subdivision, Mayo-Tsanaga Division, Far North Region, Cameroon) (11°03′ 15.22″N; 13°58″ 35.09 E; 405 m alt.). The samples were authenticated at the Herbarium of the Garoua Wildlife School by comparison with existing specimens recorded under number 6408/HEFG. Powders (200 g each) of the leaves or seeds were extracted for 1 hour with 2 L of distilled water at 70°C. After cooling and filtration, the filtrate obtained was evaporated in a ventilated oven at a temperature of 55°C to yield 9.75% and 11.50% extract residues of the leaves and seeds, respectively.

### 2.3. Animal Material

Frogs (*Bufonideae*) weighing between 40 and 65 g were kept in an artificial pond located at the Maroua Protestant College. The frogs were given free access to food throughout the acclimatization period. Their diet consisted of insects such as termites and small crickets; a vitamin complement was added to the insects before giving them to frogs. Animal procedures were conducted with strict adherence to the NIH Guide for the Care and Use of Laboratory Animals (NIH Publication #85-23, Rev. 1985).

### 2.4. Experimental Design

#### 2.4.1. Phytochemical Screening of Extracts

Qualitative phytochemical screening was carried out to check the presence of some classes of bioactive compounds contained in this extract [19].

#### 2.4.2. *In Vitro* Evaluation of the Antioxidant Potential of Aqueous Extract of the Leaves and Seeds of *D*. *metel*

The reducing power of the extracts by the FRAP method as well as the anti-free radical activity of DPPH were determined according to the methods described by Chan et al. [20] and Sun et al. [21], respectively. Briefly, the absorbance of the reaction medium was determined, and then, the antioxidant capacity of the sample was determined using a calibration range established, respectively, with ascorbic acid and Trolox (0–125 ug/mL).

#### 2.4.3. Isolated Frog Heart Preparation

The isolation of the frogs' hearts was performed following the protocol described previously by Tsala et al. [18]. The frogs were decerebrated, and the medulla was removed by pithing and then placed on a dissecting board. An incision was made at the midline of the belly and the sternum and thoracic musculature, respectively, and was split. A triangular cut was made at the level of the thorax to clear the heart entirely. The heart was then gently removed from the pericardium. The aorta was sectioned and a thread was passed underneath to fix it on the cannula. A small incision was made in the aorta to introduce the cannula filled with the physiologic solution (Ringer), which was inserted into the heart, and the surrounding tissues were delicately cut. A thin pin hook was passed through the tip of the ventricle and with the help of a fine thread attached to the hook, it was tied to the free limb of Sterling's heart lever which was fixed to a stand. A proper tension was adjusted by altering the height of the lever. The perfusion fluid in the cannula was completely displaced by Ringer containing the substances to be investigated.

#### 2.4.4. Induction of Heart Failure

Hearts were divided into 4 groups of 6 each depending on the various treatments, that is, normal, ringer, ascorbic acid, 3 mM; aqueous extract of the leaves of *D*. *metel* (AELDM), 1.5 mg/mL and aqueous extract of the seeds of *D*. *metel* (AESDM), 2.5 mg/mL. Acetylcholine was given before and after each treatment to confirm or not muscarinic receptors were physiologically active. Oxidative stress was induced according to the method previously described by Etou et al. [22]. Continuous perfusion of 1 mM of H_2_O_2_ Ringer's solution was administrated to the isolated frog's heart, followed by various treatments. The contraction force and the time required to cause cardiac arrest were recorded.

#### 2.4.5. Evaluation of Some Tissue Parameters of Oxidative Stress

Homogenates of the frog's heart were prepared by crushing 0.5 g of the heart sample in 2.8 ml of phosphate buffer solution (0.2 M, pH 7.4, pKa 7.2). The mixture was homogenized at 3000 rpm for 15 minutes at 4°C. The supernatant was recovered and malondialdehyde (MDA) concentration was evaluated using the technique described by Devasagayam et al. [23]. Superoxide dismutase (SOD) and catalase (CAT) activities were measured according to methods described by Misra and Fridovich [24] and Sinha [25], respectively.

### 2.5. Statistical Analysis

The statistical analysis of the results was performed using the GraphPad Prism 5.00 software, and the results are presented as mean ± standard error of average (ESM), for *n* = 6 hearts per group. After analyzing the variances by the one-way ANOVA test, the intergroup averages were compared using the nonparametric Tukey test. The differences were considered significant at *p* < 0.05.

## 3. Results

### 3.1. Phytochemical Screening of Extracts

The phytochemical screening of aqueous extracts of the leaves and seeds of *D. metel* revealed, respectively, the presence of flavonoids (4.31 g Eq quercetin/100 g E; 4.60 g Eq quercetin/100 g E), saponins (2.69 g Eq of galactose/100 g E; 1.83 g Eq of galactose/100 g E), alkaloids (2.1 g Eq of quinine/100 g E; 4.6 g Eq of quinine/100 g E), total phenolic compounds (16.87 g Eq GA/100 g E; 29.06 g Eq GA/100 g E), and tannins (1 g Eq of catechin/100 g E; 1.6 g Eq of catechin/100 g E).

### 3.2. *In Vitro* Antioxidant Potential of Aqueous Extract of the Leaves and Seeds of *D*. *metel*

**Figure 1:**
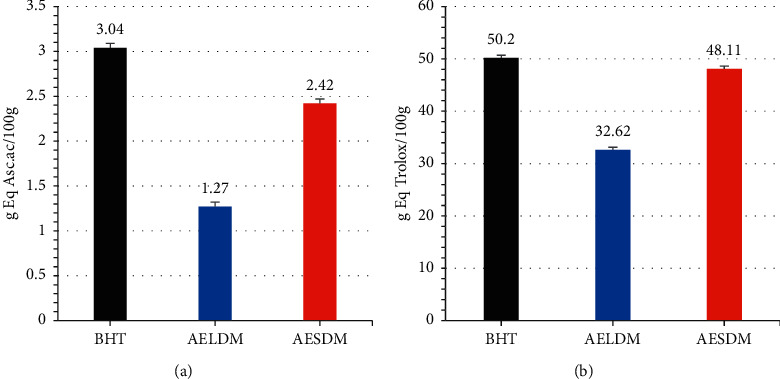
Reducing power by the FRAP method (a) and anti-free radical activity of DPPH (b) of *D*. *metel*. BHT: butilated hydroxitoliene; AELDM: aqueous extract of the leaves of *D*. *metel*; AESDM: aqueous extract of the seeds of *D*. *metel*; Asc. ac: ascorbic acid; g Eq: gram equivalent.

The aqueous extract of the seeds of *D*. *metel* has more ions Fe^3+^ reducing (2.42 Eq g of ascorbic acid/100 g extract), when compared to the aqueous extract of the leaves of *D*. *metel* (1.27 Eq g of ascorbic acid/100 g extract) (Figure 1(a)). Likewise, the aqueous extract of the seeds of *D*. *metel* has a higher anti-free radical power of 48.11 Eq g Trolox/100 g extract, compared to the aqueous extract of the leaves of *D*. *metel* which has an anti-free radical power of 32.62 Eq g Trolox/100 g extract (Figure 1(b)). However, the standard (BHT) is more ions Fe^3+^ reducing (3.04 Eq g of ascorbic acid/100 g extract) and has a higher anti-free radical power of 50.2 Eq g Trolox/100 g extract, compared to both extracts.

### 3.3. Effect of Aqueous Extract of *D*. *metel* Leaves and Seeds on the Survival Time of the Frog's Heart

**Figure 2:**
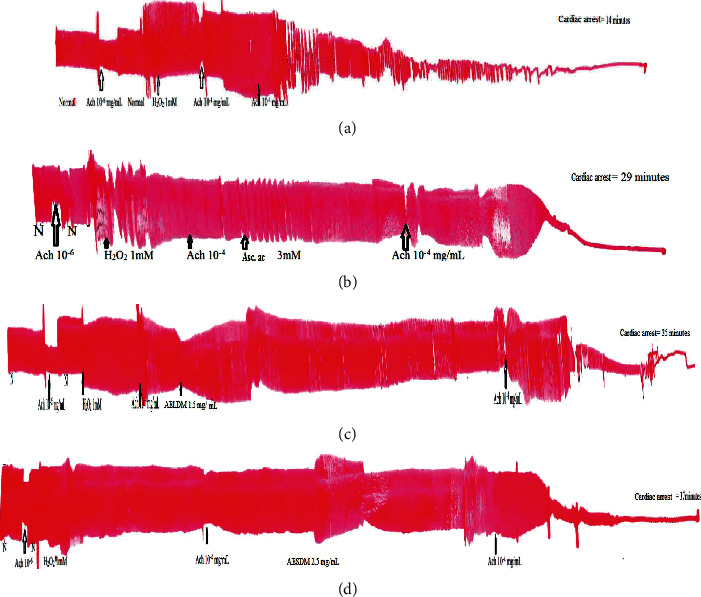
Effects of hydrogen peroxide (a), ascorbic acid (b), aqueous extract of leaves (c), and seeds (d) of *Datura metel* on the survival time of the isolated frog's hearts in a state of oxidative stress induced by hydrogen peroxide. Ach: acetylcholine; H_2_O_2_: hydrogen peroxide; Asc. ac: ascorbic acid; AELDM: aqueous extract of the leaves of *Datura metel*; AESDM: aqueous extract of the seeds of *Datura metel*.

**Figure 3:**
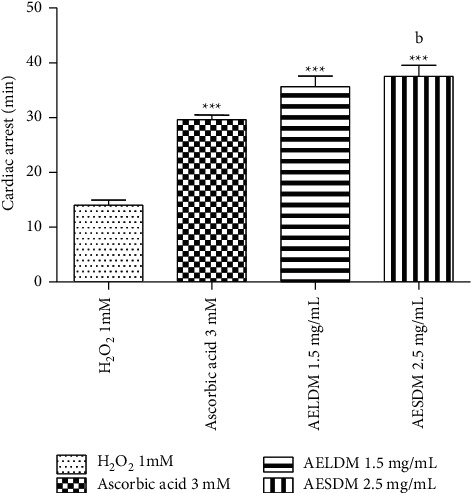
Effect of the aqueous extract of the leaves and seeds of *D. metel* on the survival time of isolated hearts of frogs in a state of oxidative stress induced by hydrogen peroxide. Each value represents the mean ± ESM, *n* = 6. ^*∗∗∗*^*p* < 0.001: statistically significant compared to the negative control (1 mM H_2_O_2_). ^b^*p* < 0.01: statistically significant compared to the positive control group (3 mM ascorbic acid). H_2_O_2_: hydrogen peroxide; AELDM: aqueous extract of the leaves of *Datura metel*; AESDM: aqueous extract of the seeds of *Datura metel*.

The survival time of the peroxidized heart survival time was 14 minutes. Administration of ascorbic acid and aqueous extracts of the leaves and seeds of *D*. *metel* (1.5 and 2.5 mg/mL) resulted in a significant increase (*p* < 0.001) in the frog heart survival time by 51.72%, 60%, and 62.16%, respectively (Figure 2). The aqueous extract of the leaves and seeds of the plant led to survival times of 35 and 37 minutes, respectively, higher than that of ascorbic acid, which is 29 minutes (Figure 3).

### 3.4. Effects of Aqueous Extract of *D*. *metel* Leaves and Seeds on Some Heart Tissue Parameters of Oxidative Stress

#### 3.4.1. Effects of Aqueous Extract of *D*. *metel* Leaves and Seeds on Malondialdehyde (MDA)

**Figure 4:**
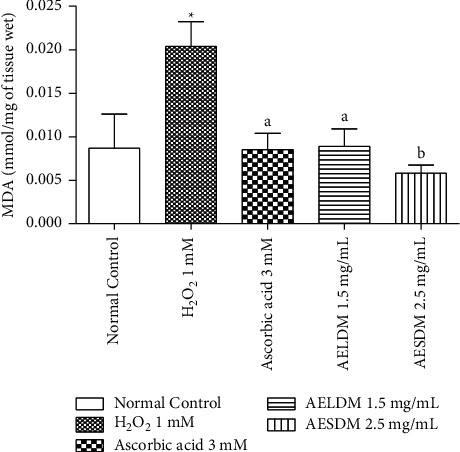
Effect of the aqueous extract of the leaves and seeds of *Datura metel* on malondialdehyde content (MDA) of the isolated frog's hearts in a state of oxidative stress induced by hydrogen peroxide. Each value represents the mean ± ESM, *n* = 6. ^*∗*^*p* < 0.05: statistically significant compared to the normal control group. ^a^*p* < 0.05 and ^b^*p* < 0.01: statistically significant compared to the negative control (1 mM H_2_O_2_). H_2_O_2_: hydrogen peroxide; AELDM: aqueous extract of the leaves of *Datura metel*; AESDM: aqueous extract of the seeds of *Datura metel*.

Perfusion of the isolated frog's hearts with 1 mM H_2_O_2_ induced a significant increase (*p* < 0.05) in MDA up to 75%, when compared to normal hearts. When peroxidized hearts were subjected to treatment with ascorbic acid, AELDM, and AESDM, the MDA levels decreased significantly (*p* < 0.05) by 30.48%, 27.27%, and 37.5%, respectively (Figure 4).

#### 3.4.2. Effects of Aqueous Extract of Leaves and Seeds of *D*. *metel* on Superoxide Dismutase (SOD) Activity

**Figure 5:**
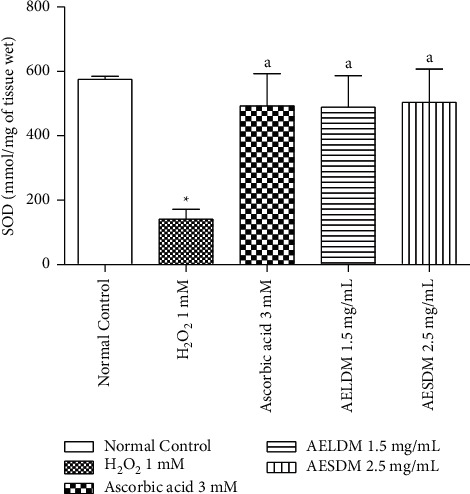
Effect of the aqueous extract of the leaves and seeds of *D*. *metel* on superoxide dismutase (SOD) of the isolated frog's hearts in a state of oxidative stress induced by hydrogen peroxide. Each value represents the mean ± ESM, *n* = 6. ^*∗*^*p* < 0.05: statistically significant compared to the normal control group. ^a^*p* < 0.05: statistically significant compared to the negative control (1 mM H_2_O_2_). H_2_O_2_: hydrogen peroxide; AELDM: aqueous extract of the leaves of *Datura metel*; AESDM: aqueous extract of the seeds of *Datura metel*.

Hearts subjected to continuous perfusion of 1 mM H_2_O_2_ showed a significant decrease (*p* < 0.05) of 20.9% in the SOD activity compared to the normal heart. However, there was a significant increase (*p* < 0.05) of the SOD activity, up to 19.44%, 21.57%, and 27.24% when hearts were formerly treated with ascorbic acid or the aqueous extract of the leaves or seeds of *Datura metel*, respectively, compared to the negative control group (1 mM H_2_O_2_) (Figure 5).

#### 3.4.3. Effects of Aqueous Extract of *D*. *metel* Leaves and Seeds on Catalase (CAT) Activity

**Figure 6:**
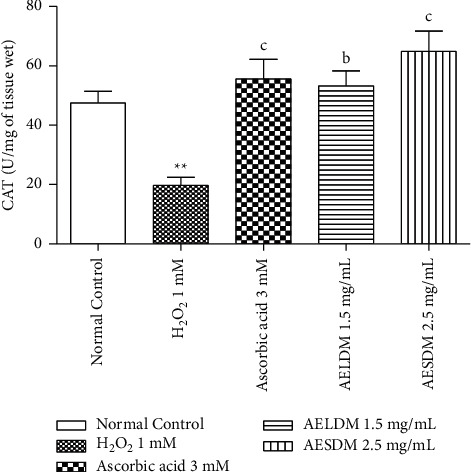
Effect of the aqueous extract of the leaves and seeds of *D. metel* on catalase activity (CAT) of the isolated frog's hearts in a state of oxidative stress induced by hydrogen peroxide. Each value represents the mean ± ESM, *n* = 6. ^b^*p* < 0.01: statistically significant compared to the normal control group. ^b^*p* < 0.01, at ^c^*p* < 0.001: statistically significant compared to the negative control (1 mM H_2_O_2_). H_2_O_2_: hydrogen peroxide; AELDM: aqueous extract of the leaves of *Datura metel*; AESDM: aqueous extract of the seeds of *Datura metel*.

Catalase activity in 1 mM H_2_O_2_ treated hearts significantly decreased (*p* < 0.05) by about 36.39%, when compared to the normal control group. After the injection of ascorbic acid, aqueous extract of the leaves, and aqueous extract of the seeds of *D*. *metel*, this activity significantly increased (*p* < 0.05) up to 41.77%, 47.93%, and 57.30%, respectively (Figure 6).

## 4. Discussion

This work aimed to evaluate the antioxidant potential of *Datura metel*, in the frog's heart failure model. The results of the phytochemical study of both parts of *D*. *metel* reveal the presence of flavonoids, saponins, alkaloids, and phenolic compounds such as tannins. These results are in agreement with the work of Kayode et al. [16].

An increase in the production of oxidizing molecules and a decrease in antioxidant defenses during heart failure have been previously described [5, 6, 26]. H_2_O_2_ is a non-radical oxygen derivative, which is toxic to cells because it allows the formation of hydroxyl radicals within cells that cause damage at the cellular level [14, 27]. When hearts are perfused with normal Ringer's solution, administration of acetylcholine causes negative inotropic and chronotropic effects, suggesting that intact muscarinic receptors have been activated by acetylcholine [28]. In the presence of H_2_O_2_, the acetylcholine-induced negative inotropic and chronotropic effects are no longer observable, due to the desensitization of muscarinic receptors, thus indicating the installation of oxidative stress at the level of the hearts [5, 26]. The present work clearly demonstrates the significant damage caused by H_2_O_2_ via ROS to cardiomyocytes. Indeed, the increased level of MDA observed in peroxidized hearts and which did not receive treatment sufficiently indicates the important degree of lipid peroxidation that has occurred in the frog's heart. It is known that malondialdehyde (MDA) is a terminal product of lipid degradation and whose content is closely related to cell membrane degradations and therefore considered as the major biomarker of oxidative stress [23, 29]. This biomarker decreases considerably in the presence of ascorbic acid and the aqueous extract of the leaves and seeds of *D*. *metel*. This important reduction in MDA content suggests that the aqueous extract of the leaves and seeds of the tested plant may have a positive effect on the lipid peroxidation induced by hydrogen peroxide, just as Ahmad et al. [29] revealed with the aqueous extracts of *Nigella sativa* seeds and *Allium sativum* as well as that of the leaves of *Rosmarinus officinalis*. Superoxide dismutase (SOD) and catalase (CAT) are essential primary antioxidant enzymes that react in the defense of the body against the toxic products of cellular metabolism. The major function of SOD is to catalyze the disproportionation of the superoxide anion to hydrogen peroxide (H_2_O_2_) and therefore reduce the toxic effects due to this free radical [30]. Catalase, on the other hand, is an enzyme that converts the hydrogen peroxide typically produced by SOD into water and molecular oxygen [31]. The results of our work revealed a significant decrease in the activity of these enzymes at the level of peroxidized hearts. However, this activity increase in the presence of ascorbic acid and aqueous extract of the leaves and seeds of *D*. *metel*. This increase in the activity of SOD and CAT could partly explain the decrease in the level of lipid peroxidation observed when the hearts were treated with the reference product and with the extract of the tested plant.

On the other hand, in our recent research, the aqueous extracts of the leaves and seed of *D*. *metel* have shown cardiotonic activity in the frog's heart [18] and cardioprotective effect on the acute cardiotoxicity induced with doxorubicin in the Wistar rats [32]. The present study aimed to investigate a possible antioxidant activity of aqueous extracts of the leaves and seeds of *D*. *metel* during H_2_O_2_-induced experimental heart failure. The data obtained showed that the oxidative stress induced by H_2_O_2_ causes irreversible cardiac arrest after the 14^th^ minute. However, in the presence of ascorbic acid and the aqueous extract of the leaves and seeds of *D*. *metel*, a significant improvement in the survival time of the hearts, and the plant extracts were more effective than ascorbic acid at the doses used in this study. Swetha et al. [6], using the same model of heart failure, demonstrated that the methanolic extract of *Leucas zeylanica* Linn leaves has antioxidant properties by improving the survival time of the isolated frog's hearts at 38 minutes. This putative antioxidant effect was confirmed by an improvement of tissue MDA content and SOD and CAT activities, and it is attributable, at least to the phenolic and alkaloid content of the extracts used [33, 34]. Several studies have shown that alkaloids, phenolic compounds, and flavonoids from plants are responsible for antioxidant properties [34, 35]. Indeed, alkaloids can act as scavengers for reactive oxygen species (antioxidants) by inhibiting lipid peroxidation and replacing the hydroxyl group with a methoxyl group [33]. Phenolic compounds in general can prevent oxidative modification by neutralizing free radicals, scavenging oxygen, or breaking down peroxides through their antioxidant activity [34].

Accordingly, the positive inotropic effect (increase in the force of contraction) without any change in the rate of contraction that was attributed to the aqueous extracts of the leaves and seeds of *D*. *metel* through a completion toward muscarinic receptor and entry of calcium in the cardiomyocyte be useful during heart failure [18]. Further studies are needed to determine if the antioxidant actions of the tested extracts directly or indirectly affect receptor functions.

## 5. Conclusion

Perfusion of the aqueous extract of the leaves and seeds of *Datura metel* succeeded to extend the survival time of the heart in the H_2_O_2_-induced isolated frog's heart failure model. This activity probably comes from their antioxidant properties. This plant could be therefore a potential source of naturally occurring agents used in the treatment of heart failure.

## Data Availability

The data used to support the findings of this study are available from the corresponding author upon request.

## References

[1] Dieng S. I. M., Fall A. D., Diatta-Badji K. (2017). Evaluation de l’activité antioxydante des extraits hydro-ethanoliques des feuilles et écorces de piliostigma thonningii schumach. *International Journal of Brain and Cognitive Sciences*.

[2] Mengong M. H., Ndjoh J. J., Nnanga N. E., Aka L. K., Bengondo M. (2021). Antioxidant activity of Aloe *Schureenfurthii* and maintenance of vitality of periodontal cells of an expelled immature permanent tooth. *Health Sciences and Disease*.

[3] Djamilatou Z. S., Djibo A. K., Seini B. S. S. H. (2021). Screening phytochimique, dosage des polyphénols et détermination de l’activité antioxydante de deux plantes antihypertensives du Niger. *European Scientific Journal ESJ*.

[4] Piba S. C., Konan P. A. K., Kone L. N. G., Kouame A. G., Kouakou R. K. D., Tra H. F. B. (2021). Phytochimie, activité antioxydante et toxicité aiguë de plantes médicinales utilisées contre les séquelles de l’accident vasculaire cérébral en Côte d’Ivoire. *International Journal of Brain and Cognitive Sciences*.

[5] Swetha B. C. H., Radhika B., Harika S. (2018). Antioxydant activity of *Paspalidium flavidum* grass using isolated frog heart. *International Journal of Pharmacy and Pharmaceutical Research*.

[6] Swetha B. C. H. (2019). Antioxydant activity of methanolic extract of leaves of *Leucas zeylanica* Linn using isolated frog heart. *Innovare Journal of Sciences*.

[7] Liu K., Liu D., Cui W. (2022). Protective effect and mechanism of traditional Chinese medicine on myocardial ischemia reperfusion injury. *Evidence-based Complementary and Alternative Medicine*.

[8] Maleki S. J., Crespo J. F., Cabanillas B. (2019). Anti-inflammatory effects of flavonoids. *Food Chemistry*.

[9] Ou J., Wang M., Zheng J., Ou S. (2019). Positive and negative effects of polyphenol incorporation in baked foods. *Food Chemistry*.

[10] Hügel H. M., Jackson N., May B., Zhang A. L., Xue C. C. (2016). Polyphenol protection and treatment of hypertension. *Phytomedicine: International Journal of Phytotherapy and Phytopharmacology*.

[11] Rahman M. M., Rahaman M. S., Islam M. R. (2021). Role of phenolic compounds in human disease: current knowledge and future prospects. *Molecules*.

[12] Bajpai V. K., Baek K.-H., Kang S. C. (2017). Antioxidant and free radical scavenging activities of taxoquinone, a diterpenoid isolated from metasequoia glyptostroboides. *South African Journal of Botany*.

[13] Daas Amiour S., Alloui-Lombarkia O., Bouhdjila F., Ayachi A., Hambaba L. (2014). Étude de l’implication des composés phénoliques des extraits de trois variétés de datte dans son activité antibactérienne. *Phytothérapie*.

[14] Praveen K. P., Satyavathi K., Prabhakar M. (2010). Antioxidant activity of traditionally used backyard Indian medicinal plants using frog heart as a model. *Research Journal of Pharmaceutical, Biological and Chemical Sciences*.

[15] Pan Y., Wang X., Hu X. (2007). Cytotoxic withanolides from the flowers of *Datura metel*. *Journal of Natural Products*.

[16] Kayode A., Imo C., Ezeonu C. S., Muhammad Z. I. (2016). Effects of ethanolic extracts of *Datura metel* on blood lipid profile of male albino rats. *International Journal of Scientific Reports*.

[17] Jakabová S., Vincze L., Farkas Á., Kilár F., Boros B., Felinger A. (2012). Determination of tropane alkaloids atropine and scopolamine by liquid chromatography-mass spectrometry in plant organs of *Datura* species. *Journal of Chromatography A*.

[18] Tsala D., Mbida H., Nnanga N., Lemba E., Habtemariam S., Ze J. (2020). Mecanism and inotropic actions of the water exracts of the leaves and seeds of *Datura metel* (*solanaceae*) on isolated frogs heart. *American Journal of Physiology, Biochemistry and Pharmacology*.

[19] Harborne J. B. (2011). *Phytochemical Methods. A Guide to Modern Techniques of Plant Analysis*.

[20] Chan E. W. C., Lim Y. Y., Wong S. K. (2009). Effects of different drying methods on the antioxidant properties of leaves and tea of ginger species. *Food Chemistry*.

[21] Sun T., Tang J., Powers J. R. (2005). Effect of pectolytic enzyme preparations on the phenolic composition and antioxidant activity of asparagus juice. *Journal of Agricultural and Food Chemistry*.

[22] Etou O. A. W., Ondélé R., Ampa R., Ngolo E., Malonga C. (2017). Evaluation des effets cardiovasculaires de l’extrait aqueux des feuilles de *Trema orientalis* (Linn.) blume (*Ulmaceae*). *Journal of Animal and Plant Sciences*.

[23] Devasagayam T. P., Boloor K. K., Ramasarma T. (2003). Methods for estimating lipid peroxidation: an analysis of merits and demerits. *Indian Journal of Biochemistry & Biophysics*.

[24] Misra H. P., Fridovich I. (1972). The role of superoxide anion in the autoxidation of epinephrine and a simple assay for superoxide dismutase. *Journal of Biological Chemistry*.

[25] Sinha A. K. (1972). Colorimetric assay of catalase. *Analytical Biochemistry*.

[26] Swetha B. C. H. (2018). Antioxydant activity of *Bauhinia blakeana* Linn leaves extract by using isolated frog heart preparation. *International Journal of Pharmacy and Biological Sciences*.

[27] Shrinivas B., Suresh R. N. (2011). Identification of *β*-carotene and *β*-sitosterol in methanolic extract of *Dipteracanthus patulus* (Jacq) nees and their role in antimicrobial and antioxidant activity. *International Journal of Phytomedicine*.

[28] Rama R. R., Jyothinath K. (2014). Effect of atropine on cardiac rhythm of frog’s heart-archived information. *Indian Journal of Basic and Applied Medical Research*.

[29] Ahmad A. K., Wessam A. R., Muna A., Abdalla I. (2017). Antioxidant and lipid peroxidation protective properties of aqueous extracts in mononuclear blood cells under high oxidants stress. *International Journal of Current Medical and Pharmaceutical Research*.

[30] Amara I. B., Hakim A., Troudi A. (2011). Protective effects of selenium on methimazole-induced anemia and oxidative stress in adult rats and their offspring. *Human & Experimental Toxicology*.

[31] Sharma P., Jha A. B., Dubey R. S., Pessarakli M. (2012). Reactive oxygen species, oxidative damage, and antioxidative defense mechanism in plants under stressful conditions. *Journal of Botany*.

[32] Mbida Hacheked H., Tsala David Emery D. E., Aboubakar Sidiki S., Amang André Perfusion A. A., Ze Minkande Jacqueline J. Z. (2020). Cardioprotective effect of the aqueous extract of seeds of *Datura metel* (*Solanaceae*) on acute cardiotoxicity induced with doxorubicin in wistar rats. *GSC Biological and Pharmaceutical Sciences*.

[33] Gan J., Feng Y., He Z., Li X., Zhang H. (2017). Correlations between antioxidant activity and alkaloids and phenols of Maca (*Lepidium meyenii*). *Journal of Food Quality*.

[34] Nijveldt R. J., van Nood E., van Hoorn D. E., Boelens P. G., van Norren K., van Leeuwen P. A. (2001). Flavonoids: a review of probable mechanisms of action and potential applications. *The American Journal of Clinical Nutrition*.

[35] Al-Waili N., Al-Waili H., Al-Waili T., Salom K. (2017). Natural antioxidants in the treatment and prevention of diabetic nephropathy; a potential approach that warrants clinical trials. *Redox Report*.

